# Iscador Qu inhibits doxorubicin-induced senescence of MCF7 cells

**DOI:** 10.1038/s41598-017-03898-0

**Published:** 2017-06-19

**Authors:** Tatjana Srdic-Rajic, Juan F. Santibañez, Ksenija Kanjer, Nevena Tisma-Miletic, Milena Cavic, Daniel Galun, Marko Jevric, Nevena Kardum, Aleksandra Konic-Ristic, Tamara Zoranovic

**Affiliations:** 1 Department of Experimental Oncology, National Cancer Research Center, Belgrade, Serbia; 2 Laboratory for Experimental Hematology and Stem Cells, Institute for Medical Research, University of Belgrade, Belgrade, Serbia; 3 Laboratorio de Bionanotecnologia, Universidad Bernardo O Higgins, General Gana 1780, 8370854 Santiago, Chile; 4 University Clinic for Digestive Surgery, Clinical center of Serbia, Belgrade, Serbia; 5 Medical School, University of Belgrade, Belgrade, Serbia; 6 Department of Surgery, National Cancer Research Center, Belgrade, Serbia; 7 Institute for Medical Research, Center of Research Excellence in Nutrition and Metabolism, University of Belgrade, Belgrade, Serbia; 8 Max Plank Institute for Infection Biology, Berlin Area, Germany

## Abstract

Chemotherapy in patients with inoperable or advanced breast cancer inevitably results in low-dose exposure of tumor-cell subset and senescence. Metabolically active senescent cells secrete multiple tumor promoting factors making their elimination a therapeutic priority. V*iscum album* is one of the most widely used alternative anti-cancer medicines facilitating chemotherapy tolerance of breast cancer patients. The aim of this study was to model and investigate how *Viscum album* extracts execute additive anti-tumor activity with low-dose Dox using ER + MCF7 breast cancer cells. We report that cotreatment of MCF7 with *Viscum album* and Dox abrogates G2/M cycle arrest replacing senescence with intrinsic apoptotic program. Mechanistically, this switch was associated with down-regulation of p21, p53/p73 as well as Erk1/2 and p38 activation. Our findings, therefore, identify a novel mechanistic axis of additive antitumor activity of *Viscum album* and low dose-Dox. In conclusion, ER + breast cancer patients may benefit from addition of *Viscum album* to low-dose Dox chemotherapy due to suppression of cancer cell senescence and induction of apoptosis.

## Introduction

Despite significant progress made in early diagnosis and treatment, breast cancer remains the most frequent cancer amongst women and the second most common cancer type in the world^1, 2^.

Hormone receptor positive breast cancers represent two thirds of all breast cancers diagnosed today^3, 4^. Despite effective targeted treatment strategies^4^ the use of chemotherapeutics is indicated for patients with primary inoperable and advanced ER/PR positive breast cancers unresponsive to first line therapy^4^. Doxorubicin (Dox), a naturally occurring anthracycline antibiotic^5^ has long been used as a chemotherapeutic component in breast cancer patient treatment^6–8^.

Antitumor activity of Dox has been assigned to induction of DNA damage and ROS production^9^. The amount of genotoxic stress and total ROS production dictates possible outcomes such as cell death or G1 and/or G2 cell cycle arrest and senescence^10–12^. Senescence induced by chemotherapeutics (therapy induced senescence, TIS) has been well studied *in vitro* and, more recently, detected in breast tumors of patients undergoing pre-operative neoadjuvant chemotherapy^13^. Though TIS has been long considered a desirable therapeutic outcome and a promising strategy in overcoming therapy resistance^13–15^, a growing body of work has indicated that TIS cells may alter response to chemotherapy^16^, escape cell cycle arrest^17, 18^ and promote tumor growth^19^ (reviewed in refs^20,21^). Most of these detrimental effects have been attributed to both autocrine and paracrine activity of senescent cell secretome designated as Senescence Associated Secretory Phenotype or SASP.

SASP components contributing to relapse and aggressive cancer occurrence^22^ include: interleukins 6 and 8 (IL-6, IL-8)^23^; amphiregulin (AREG) and growth-related oncogene (GRO) α^24, 25^, VEGF^26, 27^ or matrix metalloproteinases (MMPs)^25, 28, 29^. SASP has recently been linked to immune surveillance of damaged normal and tumor cells^30–32^. During acute normal and tumor tissue injury, one of the key SASP functions is to attract immune cells facilitating clearance of damaged senescent cells^33, 34^. However, under conditions of persistent tissue injury, damaged normal and tumor cells undergo immunoediting escaping immune surveillance^35^, an effect recently linked to SASP secretome^36^.

Therefore, compelling evidence indicates that non-cell autonomous action of SASP secretome could drive cancer relapse making eradication of therapy induced senescent cells a priority for researchers today. Complementary Alternative Medicines have long been used in oncotherapy both as therapeutic efficacy enhancers whilst facilitating tolerance of its side effects^37–44^. Phytochemical preparations of mistletoe, including aqueous extracts, are among the most frequently prescribed complementary and alternative therapies for cancer in Europe^45^. Despite well documented research and clinical studies supporting beneficial effects of Mistletoe as a complementary cancer medicine^37–44^, the most challenging obstacle towards its definitive inclusion in oncotherapy is a lack of preparation with standardized anti-tumor activity. While *Viscum Album* Extracts (VAE) exhibit potent tumor toxicity where several isolated extract compounds, such as Mistletoe Lectins (MLs), have been demonstrated to have strong apoptosis-inducing effects^46–48^. ML-induced apoptosis is primarily triggered by PI3K/Akt-, MAPK-, TLR-signalling resulting in the activation of caspases^49–51^. Its cytotoxic and anti-metastatic effect has been demonstrated in different solid tumours and leukaemia cell lines *in vitro* and *in vivo*^52–55^. MLs also display cytotoxic effects on multidrug-resistant cancer cells (e.g. *MDR* + colon cancer cells^56^ and enhance cytotoxicity of anticancer drugs^57, 58^.

Furthermore, VAEs seem to interfere with tumour angiogenesis^59, 60^. Injected into tumour-bearing animals, VAEs display growth-inhibiting and tumor reducing effects^61, 62^. Iscador Qu 5 mg Spezial represents Mistletoe aqueous extract suitable for subcutaneous injections with standardized dry substance, viscotoxin and mistletoe lectin content^63, 64^. Antitumor-activity of Mistletoe extracts has been shown to vary depending on both harvest season and host plant species^65^ allowing for designations such as Qu, M or P identifying Mistletoe hosts as Quercus (oak), Malus (Apple) or Pinus (Pine) respectively.

We have previously shown that *Viscum album/*aqueous extract (VAE) increases anti-leukemic effectiveness of doxorubicin by preventing G2/M arrest and inducing apoptosis^66^. The aim of this study was to investigate whether *Viscum album* preparation (Iscador Qu) potentiates Dox toxicity at sub-therapeutic concentrations in MCF7 ER + breast cancer cells as well as its mechanism of action.

## Results

### Isc Qu treatment of MCF7 cells abrogates low-dose Dox induced G2/M arrest

Baring in mind that low-dose chemotherapy induces cell cycle arrest and senescence, we wanted to ask the question whether Mistletoe extract (Isc Qu) anti-tumor activity targets this cell population.

**Figure 1:**
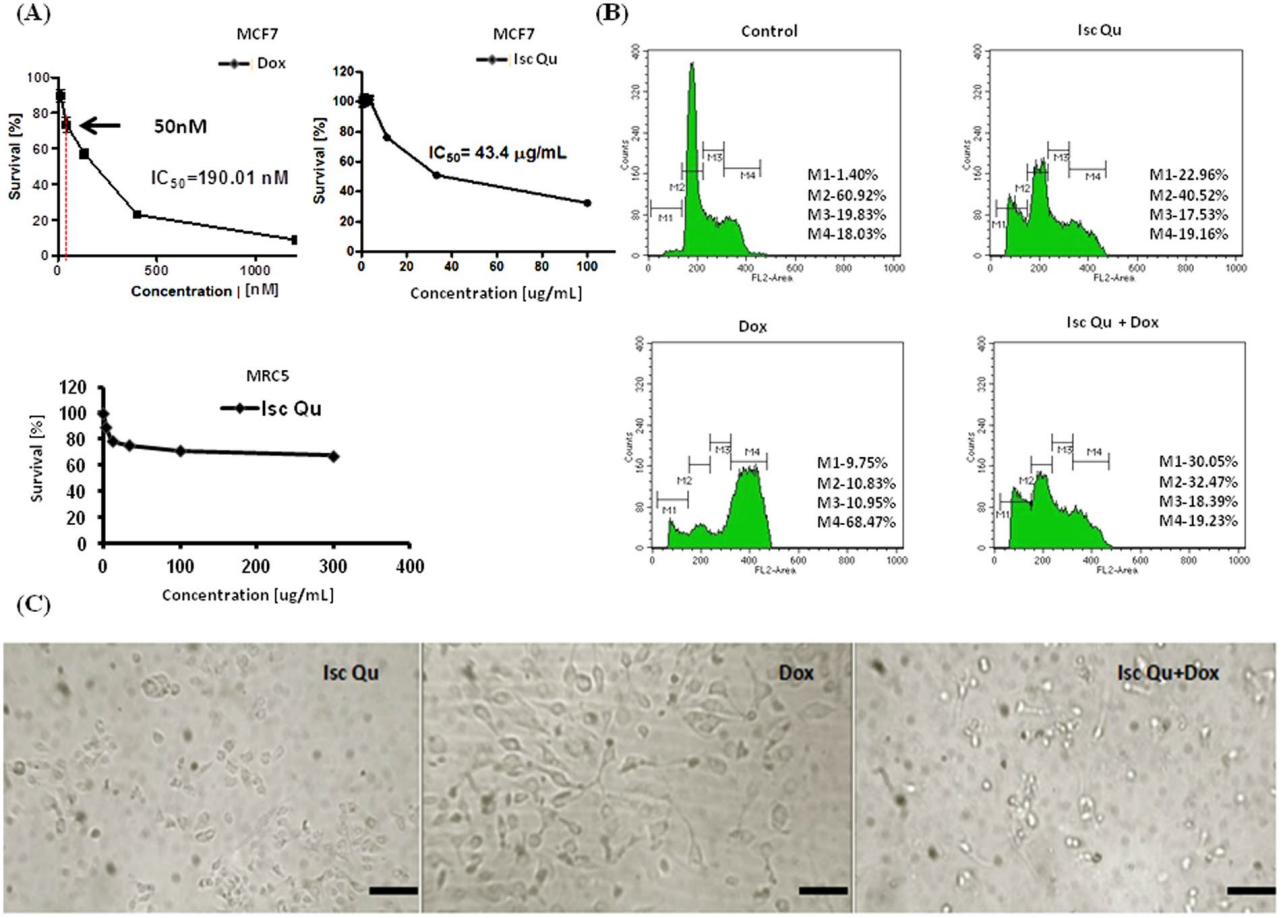
Isc Qu treatment of MCF7 cells abrogates low-dose Dox induced G2/M arrest. (**A**) MCF7 survival plots upon 72 h monotreatment with: Dox (top left), Isc Qu (top right). MRC5 survival plot upon 72 h monotreatment with Isc Qu (bottom). (**B**) Changes in cell cycle phase distribution of MCF7 cells upon 72 h treatment with Isc Qu (85 µg/mL), Dox (50 nM) or combination Isc Qu (85 µg/mL) + Dox (50 nM) are shown. After treatment, cells were stained with propidium iodide and analyzed by flow cytometry for cell cycle phase distribution changes. M1- apoptotic cells with DNA content corresponding to sub-G1 fraction; M2- cells with DNA content corresponding to G0/G1 phases; M3- cells with DNA content corresponding to S phase; M4- cells with DNA content corresponding to G2/M phases. (**C**) Light microscope photographs of morphological changes of MCF7 cells induced by Dox. Magnification, 10X.

In a series of 48 and 72 h treatments of MCF7 cells with increasing Isc Qu and Dox concentrations (Fig. S1A left and right tables, respectively), we have observed the strongest synergistic cytotoxicity after 72 h treatment with 50 nM Dox and 85 ug/mL Isc Qu (Fig. S1A). Furthermore, 72 h treatment of MCF7 cells with 50 nM Dox had the lowest impact on cell viability (Fig. 1A, top left) while potently inducing G2/M arrest (Fig. 1B). MTT assay^67, 68^ results after 72 h treatment of MCF7 cells with a range of Isc Qu concentrations revealed that IC_50_ value for Iscador Qu was 43.4 µg/mL (Fig. 1A, top right). Interestingly, 72 h treatment of MRC5 non-tumorigenic fibroblast cell line with Isc Qu did not exhibit marked toxicity even in the concentration range well above MCF7 IC50 value (300; 100; 33.3; 11.1 and 3.7 µg/mL) (Fig. 1A, bottom). Furthermore, all concentrations used for MRC5 control cell treatment seem to fall well below the IC50 mark (Fig. 1A bottom).

Thus, during 72 h cotreatment experiments with 50 nM Dox, Iscador Qu concentration of 85 µg/mL induced the strongest synergistic antitumor activity (Fig. S1A, right table). In addition, Isc Qu concentration of 85 µg/mL had no significant impact on MRC5 fibroblast viability (Fig. 1A, bottom) and was therefore selected as a minimal, most effective and well tolerated concentration.

MCF7 mono-treatment with 50 nM Dox resulted in an minor increase in subG1 population and cell death (Fig. 1B, bottom left and Fig. 1C, right) and massive expansion of G2/M arrested population from 18, 03% in untreated (Fig. 1B, top left) to 68, 47% in Dox treated group (Fig. 1B bottom left). Interestingly, cotreatment with 85 ug/mL Isc Qu abrogated Dox induced G2/M cell cycle block (Fig. 1B, bottom right) accompanied by concomitant increase of dying sub-G1 population (Fig. 1B, bottom right). Collectively, our data confirm tumor-selective Isc Qu toxicity and reveal that additive antitumor activity of Isc Qu with low-dose Dox occurs by preventing G2/M arrest and triggering cell death.

### G2/M arrest override during MCF7 cotreatment with Isc Qu and Dox activates intrinsic cell death pathway

**Figure 2:**
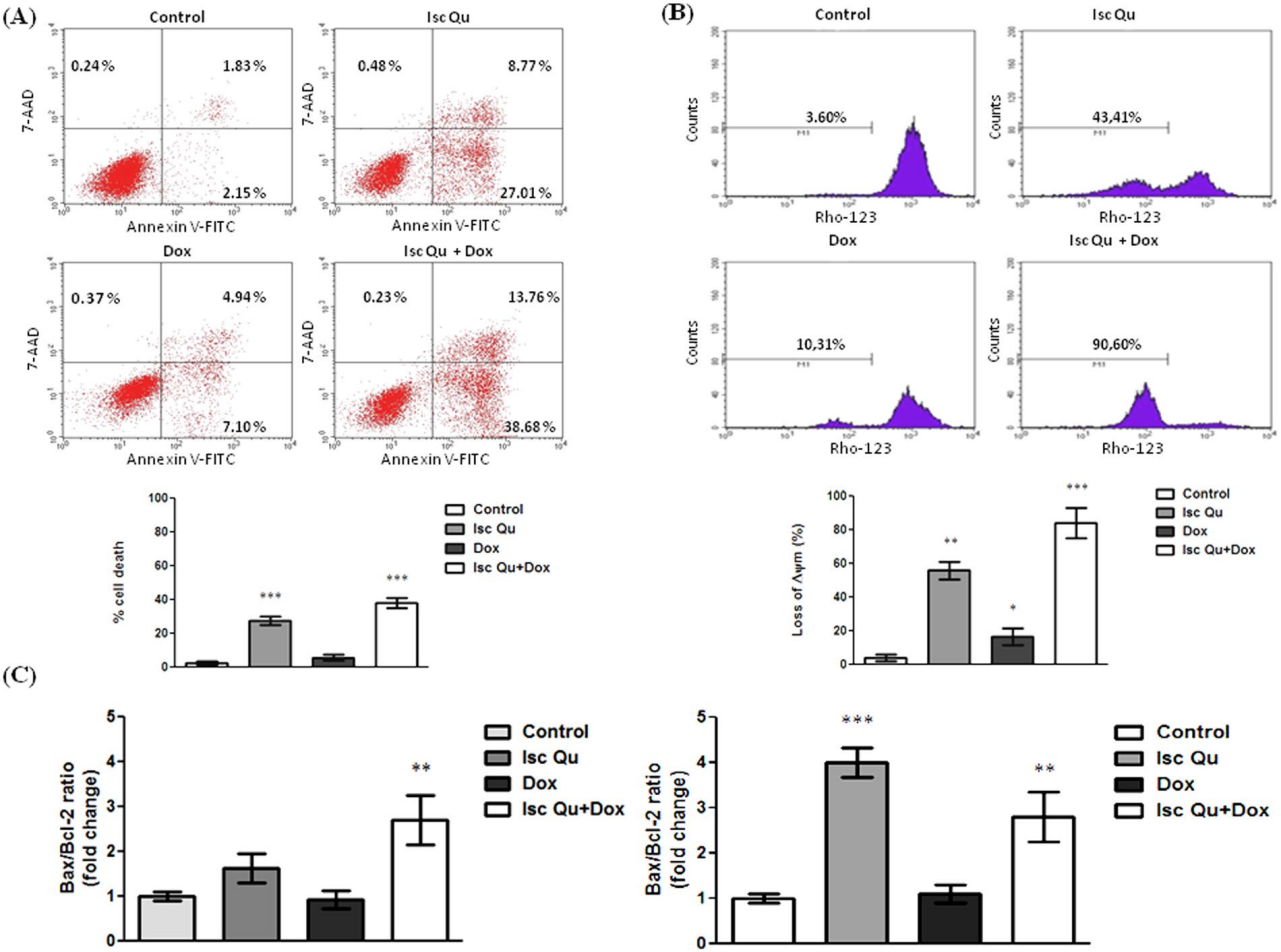
Cotreatment of MCF7 with Isc Qu and Dox activates intrinsic cell death pathway. (**A**) Proportion of early apoptotic and late apoptotic/necrotic cells was measured by the bivariate Annexin V/7AAD flow cytometry after 72 h treatment of MCF7 cells with Isc Qu (85 µg/mL), Dox (50 nM) or combination Isc Qu (85 µg/mL) + Dox (50 nM) (top). Bar graph showing results presented as the mean ± SEM of three independent experiments (bottom). (**B**) Dissipation of mitochondrial membrane potential was assessed by flow cytometry using rhodamine 123 staining (top). Bar graph showing results presented as the mean ± SEM of three independent experiments (bottom). (**C**) Bax/Bcl-2 ratio levels. Analysis of Bcl-2 and Bax protein(left) and mRNA expression (right). Detected mRNA levels were normalized to GAPDH. Results are presented as the mean ± SEM of three independent experiments. Asterisks denote statistical significance compared to control cells (*p < 0.05; **p < 0.01; ***p < 0.001). Representative dot plots from three independent experiments performed in triplicate are shown.

To gain insight into events underlying Isc Qu mediated switch from senescence to cell death of Dox treated MCF7 cells, we asked what type of cell death was prevalent under cotreatment conditions. Compared to Dox treated controls, 72 hour cotreatment of MCF7 cells with 85 µg/mL of Isc Qu and 50 nM Dox led to strong increase in Annexin V labeled early apoptotic cell population (Fig. 2A), as well as late apoptotic cells detectable by double Annexin V and 7AAD staining (Fig. 2A). Consistent with increased early and late apoptotic marker positivity (Fig. 2A), cotreatment with Isc Qu and Dox increased the detachment of MCF7 cells (Fig. 1C, right) with nearly 100% trypan blue positivity detected among cells in suspension (not shown). Cytotoxic drugs have been shown to activate intrinsic/mitochondrial apoptotic pathway^69^ reflected by measurable changes in mitochondrial membrane potential (∆ψm)^70, 71^, release of cytochrome C, induction and/or activation of proapoptotic factor Bax and effector caspase3^72–75^. FACS Rh123 fluorochrome incorporation assay^76^ revealed a dramatic loss of mitochondrial membrane potential (Δψm) in MCF7 cells upon 72 hour cotreatment with Isc Qu (Fig. 2B) indicating initiation of mitochondrial apoptotic pathway program. Moreover, FACS and qPCR analysis revealed a prominent increase of Bax/Bcl-2 ratio during cotreatment with Isc Qu compared to monotreatment conditions (Fig. 2C) confirming the activity of the apoptotic program. Thus, synergistic anti-tumor activity observed during MCF7 cotreatment with sub-apoptotic concentrations of Dox and Isc Qu is achieved by activation of intrinsic apoptotic program.

### Isc Qu cotreatment prevents low-dose Dox induced senescence and SASP in MCF7 cells

Next, we sought to address the mechanistic basis underlining Isc Qu mediated switch from Dox induced MCF7 cell senescence to apoptosis. Sub-apoptotic doses of chemotherapeutics have been shown to induce cell-cycle arrest and senescence both *in vitro* and in breast cancer patients^13, 77–80^. Considering that Dox induced G2/M accumulation of MCF7 cells was lost upon co treatment with Isc Qu (Fig. 1B), we next asked whether Isc Qu prevents accumulation of senescent tumor cells.

**Figure 3:**
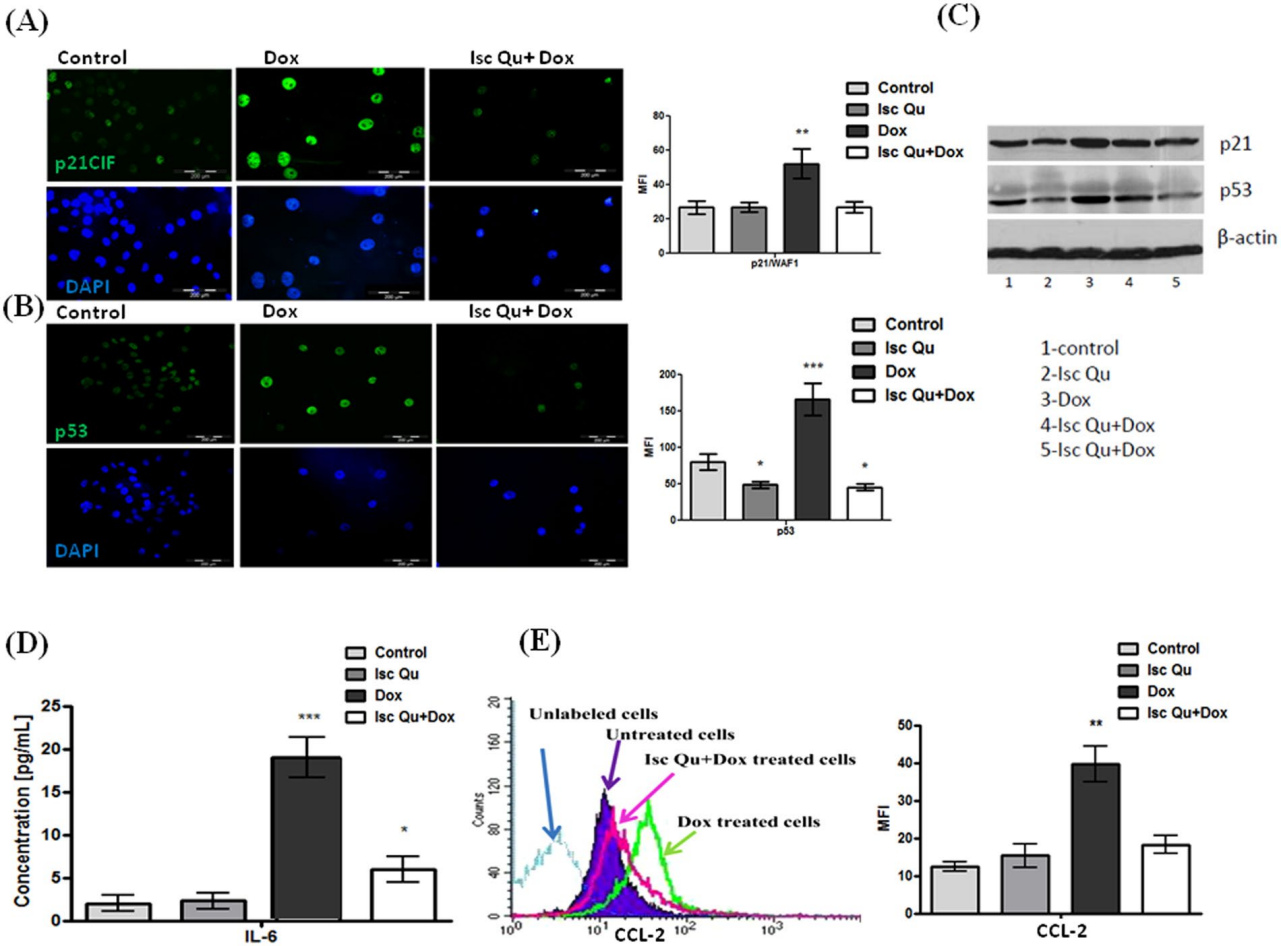
Isc Qu cotreatment prevents low-dose Dox induced senescence and SASP in MCF7 cells. Confluent MCF7 cells were treated with Isc Qu (85 µg/mL) and Dox (50 nM) alone or in combination for 72 hours and were subjected to immunofluorescence, FACS analyses and western blot. Immunofluorescent labeling of (**A**) p21 and (**B**) p53 protein expression, as well as (**C**) FACS analysis and immunobloting. Cell nuclei are labeled with DAPI (blue). (**D**) IL-6 protein levels in the supernatant of cultured MCF7 cells upon 72 h treatment with Isc Qu (85 µg/mL) and Dox (50 nM) alone or in combination, were measured using an ELISA. IL-6 activity was converted to pg according to the manufacturer’s instructions (Affymetrix, eBiosciences, CA, USA). (**E**) Protein expression of CCL-2 in treated MCF7 cells was determined by flow cytometry (left) and presented as MFI- the mean fluorescence intensity (right). Results are presented as the mean ± SEM of three independent experiments. Asterisks denote statistical significance compared to control cells (***p < 0.001). Representative photos and histogram for at least three independent experiments performed in triplicate are shown.

Compared to Dox treated controls, 72 hour cotreatment of MCF7 cells with 85 µg/mL of Iscador Qu and 50 nM Dox dramatically reduced appearance of senescent cells characterized by flattened morphology with enlarged nuclei (Fig. 1C) and SA-ß-Gal-positivity (Fig. S1B). Cancer cell response to genotoxic stress is mediated by p53/p21 and p16INK4a/pRB tumor suppressive pathways^81–87^. Both pathways are complex; each having multiple upstream and downstream regulators and modifying side branches^88, 89^. Moreover, these pathways were shown to cross-regulate each other^90–92^. Using flow cytometry, immunofluorescence staining and western blot, we next measured expression levels of both proteins under treatment conditions described. A significant increase in both p21/^WAF1^ and p53 protein was observed 72 hours after MCF7 Dox treatment (Fig. 3A–C). qPCR data revealed that gene expression of both p21 and tp53 has increased under all treatment conditions (Fig. S1C and S1D) but resulted in protein stabilization and accumulation only upon Dox treatment (Fig. 3A–C). These results show that unlike during Dox treatment, Isc Qu induced p21 promoter activity is independent of p53 likely due to absence of efficient DDR pathway activation.

SASP represents a specific program triggered by genotoxic stress in cancer cells^25^. SASP components may differ depending on the cell type, tissue type or genomic stressor^93^. Though efforts are still ongoing to identify SASP markers unique for specific cell context, IL-6 and IL-8 are among most prominently detected SASP cytokines that seem to be directly controlled by persistent DNA-damage signaling^94^. Thus, we essayed expression of IL-6 by enzyme linked immunosorbent assay (ELISA). 72 h Doxorubicin treatment of MCF7 cells resulted in a ten-fold induction of IL-6 secretion (Fig. 3D) and increased STAT3 activity (Fig. S1E) supporting previously described role for IL-6/STAT3 signaling in senescence establishment and SASP maintenance^95, 96^. Conversely, cotreatment with Isc Qu reduced IL-6 secretion (Fig. 3D) and STAT3 activation (Fig. S1E).

Though IL-6 levels detected upon Isc Qu and Dox cotreatment were higher compared to controls (Fig. 3D), STAT3 activation seems to be efficiently blocked by Isc Qu and remained significantly lower compared to untreated controls (Fig. S1E). *Viscum album* extracts have been reported to increase tumor infiltration and immune system activity^97^. Among SASP secretome components with potent adverse activity, CCL-2 has been recently described as an essential mediator of immune surveillance suppression allowing for tumor progression and metastases^13, 98, 99^. To investigate whether G2 arrested senescent cells produce CCl-2 and if this production is affected by Isc Qu cotreatment, we have assayed for CCL2 expression under described treatment conditions using FACS. Whereas 72 h Dox treatment of MCF7 cells potently induced CCL-2 protein and mRNA expression, addition of Isc Qu strongly abrogated these effects (Fig. 3E). Collectively, our results show that addition of Isc Qu to low-dose Dox efficiently blocks senescence from G2/M cell cycle arrested cells and SASP.

### Isc Qu induced G2/M senescence switch to apoptosis is accompanied by p38 and Erk1/2 inhibition

One of the well studied mechanisms of Dox toxicity in tumor cells is induction of ROS^100^. While mitochondria can generate damaging ROS^101^, mitochondrial defects were demonstrated to promote cellular senescence^102–104^. Finally, Mistletoe extracts have been reported to have a potent antioxidant activity^105–109^.Therefore, we asked whether alterations of ROS levels and corresponding signaling pathways could be responsible for the switch from senescence to apoptosis observed during IscQu cotreatment with low-dose Dox.

**Figure 4:**
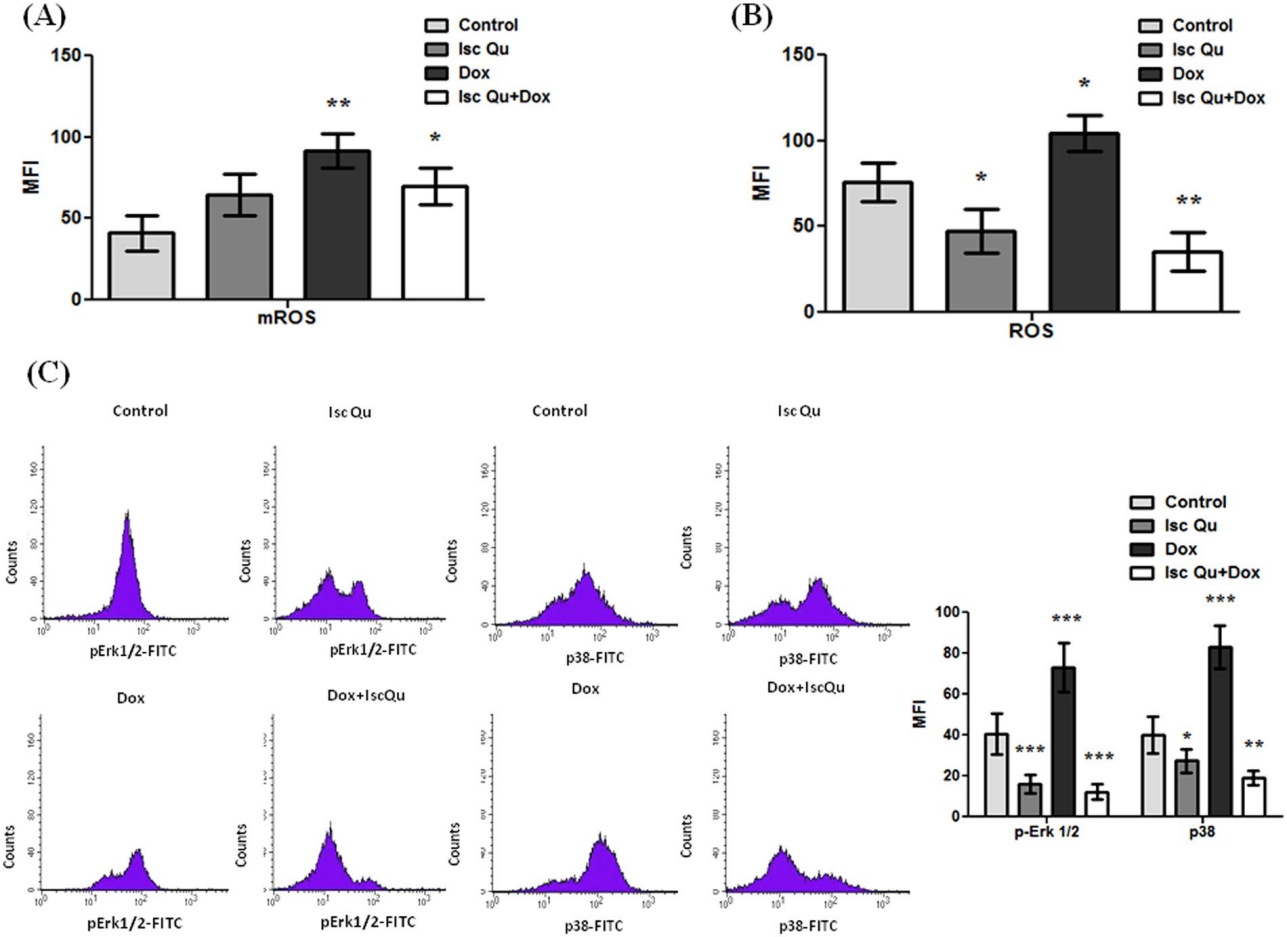
Isc Qu induced G2/M senescence switch to apoptosis is accompanied by p38 and Erk1/2 inhibition. (**A**) After 72 hour treatment with Isc Qu (85 µg/mL) and Dox (50 nM) alone or in combination, MCF7 cells where stained with dyhydrorhodamine 123 for 20 min in order to measurelevels of mitochondrial ROS, or (**B**) incubated 30 min with DCFH-DA to measure total ROS levels. (**C**) Phosphorylated pErk1/2 and p38 protein levels were assessed by flow cytometry. MFI-mean fluorescence intensity. Results are presented as the mean ± SEM of three independent experiments. Asterisks denote statistical significance compared to control cells (*p < 0.05; **p < 0.01; ***p < 0.001). Representative histograms for at least three independent experiments performed in triplicate are shown.

Data presented in Fig. 4A reveal that all treatment conditions resulted in substantial increase of mitochondrial ROS (mROS) production with maximal levels detected in cells undergoing Dox monotreatment. Interestingly, total ROS levels did not mimic alterations observed in the mitochondrial compartment though maximum total ROS production was also detected upon Dox monotreatmet (Fig. 4A and B). These results suggest that Isc Qu likely acts by suppressing ROS generation from other cellular sources such as increased signaling, peroxisome, hyperactivation of oxidases, cyclooxygenases, lipoxigenases etc.^110^.

By increasing production of distinct ROS species^111, 112^ Doxorubicin was shown to activate Erk1/2 and p38, two MAP kinases described to have a central role in establishment of senescence^113, 114^, SASP induction^115^ and survival upon oxidative injury^116, 117^. To investigate whether Isc Qu mediates a switch from senescence to apoptosis via inhibition of Erk1/2 and p38 MAP kinases, we examined the status of Erk1/2 and p38 by flow cytometry. Increased levels of both activated Erk1/2 and p-38 could be readily observed upon 72 h treatment with Dox (Fig. 4C) in line with the peak ROS production (Fig. 4A and B). Isc Qu addition almost completely abolished this effect reducing both total ROS and activated p38 well below Isc Qu mono-treatment or control levels (Fig. 4B and C, respectively). Conclusively, these data suggest that Isc Qu blocks Dox induced ROS production, consequent activation of Erk1/2 and p38 kinases preventing senescence establishment and SASP.

## Discussion

Tumor suppressor function of cellular senescence has been identified in the context of the irreversible cell cycle exit^118^ as well as autocrine and paracrine SASP mediated effects: cell cycle arrest reinforcement^119–122^ propagation of senescent phenotype on neighboring damaged cells^123^ and supporting tissue regeneration^124–126^. Moreover, SASP can elicit immune surveillance and clearance of both tumor^127–129^ and neighboring damaged cells^124^.

Paradoxically, more recent studies have demonstrated that SASP can promote tumorigenesis by supporting proliferation and invasion of surrounding tumor cells^21, 23, 130^, promoting angiogenesis^131^ or creating immunosuppressive environment allowing for tumor cell escape from immune system recognition and clearance^98, 99^.

Thus, beneficial and/or detrimental aspects of SASP appear to be critically dependent on the senescence trigger and specific SASP secretome production^132^.

Our study was designed to identify whether and how *Viscum album* extracts could potentiate anti-tumor activity of sub-apoptotic Doxorubicin concentrations in MCF7 breast cancer cells. In this study we report for the first time that VAE potently inhibits chemotherapy induced senescence and triggers apoptosis. We have also confirmed previous reports describing selective anti-tumor toxicity of VAE/Isc Qu^66^. We show that 72 h MCF7 cell treatment with sub-apoptotic Dox concentrations induces G2/M cell cycle arrest, senescence and SASP which were blocked by cotreatment with Isc Qu. The switch from G2/M senescence entry into apoptosis seems to be regulated by preventing Dox mediated ROS accumulation and Erk1/2 p38 activation. Our data suggest that G2/M cell cycle exit and senescence are regulated by both p53-p21 and p38 activated INK4A/Rb pathway probably owing to their extensive crosstalk. A trigger stimulus for Dox induced G2/M senescence seems to be moderate ROS production leading to sustained p38 activation^114, 133^. Persistent p38 activity was shown to induce SASP^96, 116^ largely via facilitating NF-kB activation and its recruitment to inflammatory gene promoters such as IL-6 and CCL-2^134^. Previous work has shown that IL-6 plays a key role in oncogene induced senescence establishment^25^ and inflammatory secretome maintenance^135^. Thus, Increased ROS production during Dox treatment would likely trigger p38 activity^136^ establishing a positive feedback loop in IL-6 secretion and secretome maintenance. We report that additive antitumor activity of low-dose Dox (50 nM) with Iscador Qu triggers apoptosis while reducing ROS, p53 and p21 levels. Our results mimic the findings of Delebinski and colleagues in lauekaemia cells^137^ where mistletoe lectins where shown to suppress p53 and induce TRAIL expression and apoptosis in a dose dependent manner. It is possible that, as previously shown^138–140^, low-dose-Dox treatment sensitizes MCF7 cells to Mistletoe lectin-induced TRAIL expression and apoptosis while ROS quenching by the extract attenuates p38, Erk1/2, p53 and p21 activation. Considering that we haven’t analyzed temporal profiles of ROS production as well as Erk1/2 and p38 activity, it is not clear whether putative cross-talk with TRAIL signaling and resulting apoptotic shift takes place at the onset of p38 activation or establishment of the positive feedback loop mediated by inflammatory signaling. To our knowledge, this is the first report showing that *Viscum Album* extract could prevent chemotherapy induced senescence by redirecting cells into apoptosis. Further studies elucidating the exact timing and molecular mechanism of this “switch” are of special interest both for the therapeutic purposes and prevention of ageing-related diseases. In conclusion, our data indicate that Isc Qu can sensitize MCF7 cells to apoptotic cell death at senescent concentration of the Dox by down-regulation of ROS, p21/^WAF1^, and p53 and by suppressing SASP profile through prevention of activation of Erk1/2, p38 and STAT3. Therapeutic interventions with Isc Qu applied as CAM, whether by dampening the SASP profile or by preventing the therapeutic senescence through induction of apoptotic cell death, should be much more effective. Whether Isc Qu is effective in targeting endogenously senescent tumor or non-tumorigenic cells remains to be addressed.

## Materials and Methods

### Drug

Doxorubicin hydrochloride (Sigma-Aldrich, St. Louis, USA) was diluted in sterile water. For the purpose of the study, we used Iscador Qu Spezial (Iscador AG, Switzerland) in 1 mL ampules of 5 mg. This preparation is fermented aqueous extracts of mistletoe plants growing on oak trees. As confirmed with the manufacturer, Iscador Qu Spezial batches used in our experiments are standardized on the basis of their contents of mistletoe lectins 391 ± 18.3 ng/mL and viscotoxins 13 ± 3 µg/mL.

### Cell line

All cell lines used in this study were obtained from the American Type Culture Collection (Rockville, MD) unless specified otherwise. Human breast adenocarcinoma cells (MCF-7) and a single human normal cell line (MRC-5) were maintained as monolayer culture in a nutrient medium, MRC-5 cells in the Roswell Park Memorial Institute (RPMI) 1640 medium, while MCF7 cells in the DMEM medium. Powdered RPMI 1640 medium, and DMEM modified medium, were purchased from Sigma Chemicals Co, USA. Nutrient medium RPMI 1640 was prepared in sterile deionized water, supplemented with penicillin (192 U/mL), streptomycin (200 µg/mL), 4-(2-hydroxyethyl) piperazine-1-ethanesulfonic acid (HEPES) (25 mM), L-glutamine (3 mM) and 10% of heat-inactivated fetal calf serum (FCS) (pH 7.2). Modified DMEM nutrient medium was prepared in sterile deionized water, supplemented with penicillin (192 U/mL), streptomycin (200 µg/mL) and 10% of heat-inactivated FCS. Cells were grown at 37 °C in 5% CO2 and humidified air atmosphere, by twice weekly subculture.

### Cytotoxicity assay

Cytotoxic activity of Isc Qu and Dox on MCF7 and MRC5 cells was assessed using the MTT assay^67, 68^. After treatment in 96-well plates, 20﻿ µ﻿L of﻿ MTT solution (3-(4, 5-dimethylthiazol-2-yl)-2, 5-dyphenyl tetrazolium bromide) (Sigma-Aldrich, St. Louis, USA) was added to each well. Samples were incubated for 4 h, followed by the addition of 100 μL of 10% SDS and incubated at 37 °C. Absorbance at 570 nm was measured the next day.

Cell survival (%) was calculated as an absorbance (A570 nm) ratio between treated and control cells multiplied by 100. IC_50_ was defined as the concentration of the agent that inhibited cell survival by 50% compared to the vehicle control.

### Flow-cytometric analysis of cell cycle phase distribution

Briefly, 2 × 10^5^ cells/Petri dish (dimensions 60 × 15 mm, NUNC) were treated with investigated drugs as indicated. After collection, cells were fixed with ethanol and stained with propidium iodide (PI) (Sigma-Aldrich, St. Louis, USA)^141^. Cell cycle phase distribution was analyzed by FACS Calibur Becton Dickinson flow cytometer using Cell Quest computer software (Becton Dickinson, Heidelberg, Germany)

### Apoptotic assay

Apoptotic rates were assessed with flow cytometry using the Annexin V–fluorescein isothiocyanate/and 7AAD (BD Pharmingen, San Diego, CA, USA). Samples were prepared according to manufacturer’s instructions. Flow cytometry analysis was performed using a FACS-Calibur cytometer using Cell Quest computer software (Becton Dickinson, Heidelberg, Germany).

### Quantification of mitochondrial transmembrane potential

Mitochondrial transmembrane potential (Δψm) was measured using a cationic fluorochrome Rodamine 123 (Rh123) (Sigma-Aldrich, St. Louis, USA) as described by Yan *et al*.^76^. Briefly, 1 × 10^6^ cells resuspended in 200 μL of phosphate buffered saline were stained with Rh123 (2.5 μg/mL) for 30 min at 37 °C. After washing, samples were analyzed by flow cytometry using Cell Quest software (Becton Dickinson, Heidelberg, Germany).

### Intracellular staining

Cells stained for FACS analysis were treated as described above. For intracellular staining the following antibodies were used: mouse anti-p21^WAF1^, clone CP74 (5 µg/mL, Merck Millipore, Darmstadt, Germany), mouse anti-Bax, clone 3/Bax (1:100, BD Pharmingen, San Diego, CA, USA), FITC-conjugated monoclonal Bcl-2 antibody, clone Bcl-2/100 (1:100, BD Pharmingen, San Diego, CA, USA), mouse anti-human Phospho-p44/42 MAPK (Erk1/2) (Thr202/Tyr204) antibody (1:200, Cell Signaling, Danvers, MA, USA), mouse anti-human phospho-p38 MAPK (Thr180/Tyr182) antibody, clone 28B10 (1:800, Cell Signaling, Danvers, MA, USA), mouse anti-p53 antibody, clone DO7 (dilution 1:100, Dako, Glostrup, Denmark) and mouse anti-p73, clone ER-13 (5 µg/mL, Merck Millipore, Darmstadt, Germany).

Briefly, cells (5 × 10^5^ cells/flask 25 cm^2^) were allowed to adhere for 24 h in standard conditions, and then treated as described in Results. After the stimulation period, cells were fixed the cells immediately by adding pre-warmed Cytofix Buffer for 10 to 12 minutes at 37 °C, and washed twice with PBS containing 1% BSA. After permeabilization of the cells using of Perm Buffer for 20 minutes at room temperature and washing, cells were incubated with antibodies at room temperature for 60 minutes protected from light, and washed twice with PBS containing 1% BSA. After appropriate incubation, cells were washed three times with PBS containing 1% BSA and incubated with the corresponding FITC-coupled secondary antibodies (dilution 1:100, BD Pharmingen, San Diego, CA, USA). Samples were analyzed on a FACS-Calibur cytometer using Cell Quest software (Becton Dickinson, Heidelberg, Germany).

### Immunofluorescence Labeling

Cells (5 × 10^4^ cells/cover slip) were allowed to adhere for 24 h in standard conditions, and then treated as described in Results. Immunofluorescent labeling was performed as previously described^142^. Briefly, cells were fixed in 4% paraformaldehyde, permeabilized in 0.1% Triton-X100 (Sigma Aldrich), and then immunolabeled with primary antibodies: mouse anti-p21^WAF1^, clone CP74 (5 µg/mL, Merck Millipore, Darmstadt, Germany), mouse anti-p53, clone DO7 (dilution 1:100, Dako, Glostrup, Denmark). Samples incubated in 1% BSA in PBS served as negative control. After appropriate incubation (indicated in Results), cells were washed three times with PBS and incubated with the corresponding FITC-coupled secondary antibodies (dilution 1:100, from Pharmingen) and 1 µg/mL of DAPI (Sigma-Aldrich, St. Louis, USA) for nucleus labeling for 1 h at room temperature. Mounted cells were analyzed using an epifluorescent microscope (Olympus, Japan).

### Analysis of gene expression by Real-time PCR

Total RNA was isolated using TRI REAGENT® BD kit (Sigma-Aldrich, St. Louis, USA). cDNA synthesis from total RNA using random primers and MultiScribe™ Reverse Transcriptase from High-Capacity cDNA Reverse Transcription kit (Applied Biosystems, CA, USA). All target transcripts were detected using TaqMan® Gene Expression Assays Applied Biosystems, CA, USA. All reactions were performed in duplicate and the data represent mean ± SEM of three independent experiments. Results were analyzed using the classical delta-delta-Ct method. GraphPad Prism 5.04 (GraphPad Software, CA, USA) was used to compare means by two way analysis of variance (ANOVA) and Bonferroni method to adjust the p-value for multiple comparisons. Differences were considered significant if p < 0.05. P-values for each analysis are indicated in Fig. legends.

### Senescence associated β-galactosidase staining

Cells (2 × 10^5^ per well) were seeded into 6-well plates in DMEM culture medium and after 24 hours treated with investigated drugs for 72 hours. SA-β-gal staining was performed using Senescence β-galactosidase staining kit (Cell Signaling) according to the manufactures’ instructions. Briefly, cells were fixed for 15 min in fixative solution, washed and incubated at 37 °C with X-gal (1 mg/mL), dissolved in a solution containing 40 mM citric acid pH 6.5, 5 mM potassium ferrocyanide, 5 mM potassium ferricyanide, 150 mM NaCl and 2 mM MgCl2. After 24 h incubation, photographs were taken using a standard light microscope (Olympus, Japan).

### Enzyme-linked immunosorbent assay

MCF7 cells (5 × 10^3^ per well) were plated in 96-well plates and allowed to adhere for 24 h in standard conditions. Upon adherence, cells were treated with investigated drugs for 72 hours and the culture supernatants were collected. Supernatant concentrations of IL-6, TNF-α and TGF-β were measured using ELISA according to manufacturer’s protocols (Affymetrix, eBiosciences, CA, USA).

### Measurement of mitochondrial reactive oxygen species

MCF7 mitochondrial reactive oxygen species (ROS) levels were measured using a ROS sensitive fluorescent probe, 1, 2, 3 dihydrorhodamine (DHR). Fluorescent marker dihydrorhodamine 123 was purchased from Molecular Probes, prepared as a 5 mM stock solution in dimethyl sulfoxide, and used at 1 μM. Briefly, cultured MCF7 cells were treated with Isc Qu, Dox and their combination with untreated cells maintained as controls. Following 72 h incubation, cells were harvested, washed twice, resuspended in 1 μM DHR and incubated at 37 °C for 20 min in the dark. Levels of intracellular ROS were examined with flow cytometry (FACS Calibur, BD Biosciences, USA). Excitation wavelength was 485 nm with peak emission measured at 530 nm. Data acquisition and analyses were carried out using Cell Quest software (BD Biosciences, USA).

### Measurement of total intracellular reactive oxygen species

Generation of reactive oxygen species (ROS) in MCF7 cells was measured using a ROS sensitive fluorescent probe, 2,7-dichlorodihydrofluorescein diacetate (DCFH-DA). This probe can be oxidized to 2′,7′-dichlorofluorescein (DCF) by ROS and exhibits increased green fluorescence intensity. Briefly, cultured MCF7 cells were treated with Isc Qu, Dox and their combination with untreated cells maintained as controls. Following 72 h incubation, cells were harvested, washed twice, re-suspended in 10 mM DCFH-DA and incubated at 37 °C for 30 min in the dark. Levels of intracellular ROS were examined using flow cytometry (FACS Calibur, BD Biosciences, USA). Excitation wavelength used in measurements was 485 nm with peak emission measured at 530 nm. Data acquisition and analyses were carried out using Cell Quest software (BD Biosciences, USA).

### Western blot analysis

Following 72 h treatment, protein samples extracted from MCF7 cells were used for immunoblot assay. Cells were washed twice with ice-cold PBS, and lysed in Cell Lysis Buffer (20 mM Tris pH 7.5, 150 mM NaCl, 1 mM EDTA, 1 mM EGTA, 1% Triton X-100, 2.5 mM sodium pyrophosphate, 1 mM b-glycerolphosphate, 1 mM Na3PO4, 1 mg/mL Leupeptin, 1 mM PMSF). Cell lysates were centrifuged then normalized by the Lowry assay; proteins in lysates were separated by sodium dodecyl sulfate (SDS)- polyacrylamaide gel electrophoresis (PAGE). Proteins were transferred to nitrocellulose membranes and membranes were blocked with TBST containing 5% non-fat milk. Following overnight incubation of membranes with primary antibodies at 4 °C, membranes were washed with TBST containing 0.1% Tween 20, and incubated with goat antimouse peroxidase- conjugated secondary antibodies for 60 min at room temperature. Blots were probed with an enhanced chemiluminescence (ECL) substrate (Pierce, Thermo scientific) and exposed to Hyperfilm ECL to visualize the immunoreactive bands.

### Statistical analysis

Data are presented as mean standard deviation (SEM) of three independent experiments. Comparisons between two groups were performed using Student’s t-test. p-value 0.05 was considered to be significant.

## Electronic supplementary material

Supplementary Information
