# Author Correction: Iscador Qu inhibits doxorubicin-induced senescence of MCF7 cells

**DOI:** 10.1038/s41598-018-31450-1

**Published:** 2018-10-26

**Authors:** Tatjana Srdic-Rajic, Juan F. Santibañez, Ksenija Kanjer, Nevena Tisma-Miletic, Milena Cavic, Daniel Galun, Marko Jevric, Nevena Kardum, Aleksandra Konic-Ristic, Tamara Zoranovic

**Affiliations:** 1 Department of Experimental Oncology, National Cancer Research Center, Belgrade, Serbia; 2 Laboratory for Experimental Hematology and Stem Cells, Institute for Medical Research, University of Belgrade, Belgrade, Serbia; 3 Laboratorio de Bionanotecnologia, Universidad Bernardo O Higgins, General Gana 1780, 8370854 Santiago, Chile; 4 University Clinic for Digestive Surgery, Clinical center of Serbia, Belgrade, Serbia; 5 Medical School, University of Belgrade, Belgrade, Serbia; 6 Department of Surgery, National Cancer Research Center, Belgrade, Serbia; 7 Institute for Medical Research, Center of Research Excellence in Nutrition and Metabolism, University of Belgrade, Belgrade, Serbia; 8 Max Plank Institute for Infection Biology, Berlin Area, Germany

Correction to: *Scientific Reports* 10.1038/s41598-017-03898-0, published online 19 June 2017.

The original version of this Article contained errors in the spelling of the authors Tatjana Srdic-Rajic, Juan F Santibañez, Ksenija Kanjer, Nevena Tisma-Miletic, Milena Cavic, Daniel Galun, Marko Jevric, Nevena Kardum, Aleksandra Konic-Ristic and Tamara Zoranovic, which were incorrectly given as T. Srdic-Rajic, J. F. Santibañez, K. Kanjer, N. Tisma-Miletic, M. Cavic, D. Galun, M. Jevric, N. Kardum, A. Konic-Ristic & T. Zoranovic respectively.

These errors have now been corrected in the PDF and HTML versions of the Article, and in the accompanying Supplementary Information file.

