# Supplementary material for: Iscador Qu inhibits doxorubicin-induced senescence of MCF7 cells
Source: Sci Rep. 2017 Jun 19;7:3763. doi: 10.1038/s41598-017-03898-0 (PMC5476621; doi:10.1038/s41598-017-03898-0)
Supplement: Supplementary file 1 — Supplementary Information [file 41598_2017_3898_MOESM1_ESM.pdf]

Supplementary Information:

Iscador Qu inhibits doxorubicin-induced senescence of MCF7 cells

Tatjana Srdic-Rajic[1], Juan F Santibañez[2,3], Ksenija Kanjer[1], Nevena Tisma-Miletic[1], Milena Cavic[1], Daniel Galun[4,5], Marko Jevric[6], Nevena Kardum[7], Aleksandra Konic-Ristic[7], Tamara Zoranovic[1,8]

[1]Department of Experimental Oncology, National Cancer Research Center, Belgrade, Serbia
[2]Laboratory for Experimental Hematology and Stem Cells, Institute for Medical Research, University of Belgrade, Serbia
[3]Laboratorio de Bionanotecnologia, Universidad Bernardo O Higgins, General Gana 1780, 8370854 Santiago, Chile
[4]University Clinic for Digestive Surgery, Clinical center, Belgrade, Serbia
[5]Medical School, University of Belgrade, Belgrade, Serbia
[6]Department of Surgery, National Cancer Research Center, Belgrade, Serbia
[7]Institute for Medical Research, Center of Research Excellence in Nutrition and Metabolism, University of Belgrade, Serbia
[8]Max Plank Institute for Infection Biology, Berlin Area, Germany

Corresponding author
Dr. Tamara Zoranovic

zoranovic@mpiib-berlin.mpg.de

tamara.zoranovic@yahoo.com

Supplementary Fig. 1. Isc Qu prevents Dox induced cell cycle arrest and senescence

Synergistic effects of Isc Qu and Dox in a tested range of concentrations evaluated after 48h (A, left) and 72h (A, right) treatment. The strongest synergistic tumor cell toxicity (red box) was observed after 72h co-treatment at indicated concentrations (A, right). Morphological changes such as enlarged and flattened cell shapes accompanied with finely granular and more abundant foamy cytoplasm are characteristic features of senescent phenotype. Staining for SA-β-gal after 72 hours of drugs treatment, was performed as described in Materials and methods (B). Gene expression levels of p21 (C) and TP53 (D) were determined by qPCR, while protein expression level of pSTAT3 was assessed by flow cytometry (E). The mRNA levels were normalized to GAPDH. MFI is the mean fluorescence intensity.

All results are presented as a mean ± standard error of the mean of three independent experiments. Asterisks denote statistical significance compared with control cells (*p <0.05; **p <0.01; ***p <0.001). Representative photos for at least three independent experiments performed in triplicate are shown.

(A)

	Drug concentration (48h treatment)			
Substance	I	II	III	IV
Isc QU	21.44 ± 1.5	49.35 ± 2.0	60.87 ± 2.3	75.05 ± 2.1
Dox	10.35 ± 0.9	11.5 ± 1.2	22.55 ± 1.6	53.62 ± 2.9
Isc Qu+Dox	22.19 ± 2.0	59.33 ± 2.8	69.78 ± 2.6	72.04 ± .3.0
CI	1.410	0.723	0.843	1.614
Interaction	--	+ +	+ +	---

Isc Qu (μg/mL): I=21.25; II=45; III=85; IV=170
Dox (μM): I=0.0125; II=0.025; III=0.05; IV=0.1

	Drug concentration (72h treatment)			
Substance	I	II	III	IV
Isc QU	23.44 ± 1.8	52.35 ± 2.2	75.87 ± 2.7	80.05 ± 3.1
Dox	15.35 ± 1.1	25.01 ± 1.7	27.55 ± 1.4	60.36 ± 2.9
Isc Qu+Dox	27.19 ± 2.3	65.33 ± 2.8	85.78 ± 2.7	82.04 ± 3.2
CI	1.426	0.753	0.539	1.358
Interaction	--	+ +	+ + +	--

Isc Qu (μg/mL): I=21.25; II=45; III=85; IV=170
Dox (μM): I=0.0125; II=0.025; III=0.05; IV=0.1

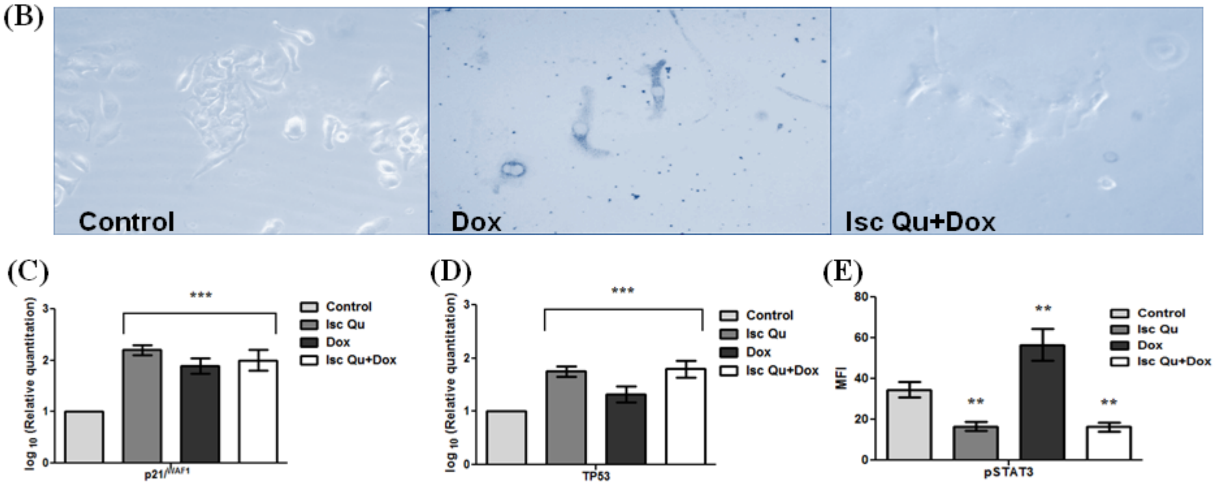

Fig. S1
